# Alcoholics anonymous and recovery in Türkiye: A qualitative study in the context of Social Identity Theory

**DOI:** 10.1111/bjso.70083

**Published:** 2026-04-10

**Authors:** Fatih Kucur, Özge Kelebek, Melek Rabia Kapıcı, Esme Temiz

**Affiliations:** ^1^ Department of Social Work, Faculty of Health Sciences Istanbul University‐Cerrahpasa Istanbul Türkiye; ^2^ Department of Social Work, Faculty of Health Sciences Anadolu University Eskişehir Türkiye

**Keywords:** alcohol use disorder, alcoholics anonymous, recovery, Social Identity Theory, social networks

## Abstract

Alcoholics anonymous (AA) groups play a central role in facilitating the transition from an ‘addicted’ identity to a ‘recovering’ identity; however, empirical research on how this identity transformation is socially constructed, maintained and questioned within group contexts is limited. Drawing on Social Identity Theory (SIT), this qualitative study examines how AA group dynamics shape members' sense of belonging, processes of identity reconstruction and experiences of recovery while also illuminating the vulnerabilities that may destabilize recovery identity. The study involved in‐depth interviews with 20 AA members in Istanbul who had participated in meetings for a minimum of 6 months. The data were analysed using reflexive thematic analysis. The findings were organized into three interrelated themes: (1) AA as an in‐group: the construction of a recovery identity, (2) reinforcement of the recovery identity through social interactions and (3) vulnerabilities and threats to the recovery identity. Overall, this study demonstrates that the identity of recovery within AA is a continuous negotiation process shaped by group‐based interactions and identity threats. By highlighting the social and relational dimensions of recovery, this study extends SIT to mutual aid groups and emphasizes the central role of collective identity processes in sustaining long‐term recovery.

## INTRODUCTION

Alcohol use disorder (AUD) is a chronic, recurrent disorder characterized by loss of control over alcohol consumption and the emergence of a negative emotional state when alcohol is not consumed (Koob et al., 2020). Individuals with AUD may exhibit increasing drinking patterns that can lead to serious consequences for their physical, mental and social health (Carr et al., 2024). The risk of various psychiatric disorders (Puddephatt et al., 2022), infectious diseases (Morojele et al., 2021), chronic diseases (Hacker, 2024) and neurological diseases (Rao & Topiwala, 2020) also increases with AUD. This significantly affects the quality of life of individuals. Moreover, some cases can result in death. However, it is important to note that alcohol‐related diseases and injuries can occur even in the absence of AUD. According to the World Health Organization's (2024) report, approximately 400 million people worldwide, or 7% of the global population aged 15 and older, have an AUD. A total of 209 million people suffer from alcohol dependence. In 2019, approximately 2.6 million deaths were attributed to alcohol consumption (World Health Organization, 2024). These data alone demonstrate the impact of alcohol dependence on global health issues and the necessity of implementing active policies aimed at reducing alcohol‐related harms through treatment processes.

It is established in the literature that AUDs are primarily rooted in a combination of genetic, psychological and environmental factors (Choi et al., 2025; Obeid et al., 2020). Various screening strategies, brief interventions (Kim & Hendershot, 2020), pharmacological treatments (Fairbanks et al., 2020), psychotherapy and behavioural interventions (Hallihan et al., 2024) are among the many treatment methods used for the treatment of AUD. In addition, psychological and environmental support systems can positively influence the treatment of AUD (Gerdner & Holmberg, 2000). Many individuals who develop AUD or have milder alcohol‐related problems (subclinical) try to solve their problems either on their own, with professional support or by joining mutual aid groups (Tucker, 2020). The alcoholics anonymous (AA) group, which we address in this study, is one such support group.

Mutual aid groups such as AA provide an alternative platform that reaches millions of individuals worldwide, structuring recovery through social support, spirituality and shared experiences (Alcoholics Anonymous, 2025). Consequently, understanding how these support networks facilitate the recovery process represents a crucial area of inquiry for both clinical practice and theoretical frameworks.

### Alcoholics anonymous

AA was founded in the United States in 1935 as a 12‐step program and self‐help group based on spirituality and social support to assist individuals with AUD in their recovery. It is estimated to have over two million members in 180 countries worldwide (Alcoholics Anonymous, 2025). The first AA meeting in Türkiye was held in 1988. Although precise membership figures cannot be determined from official records, the number of AA members in Türkiye is thought to be close to 1000 (Doğancı, 2024).

The only requirement for AA membership is a willingness to quit alcohol consumption. There are no dues or fees, and members meet weekly, but no membership list is maintained. Members are encouraged to progress positively in their recovery processes by sharing experiences. This facilitates social interaction among group members (Behrendt & Burke, 2023). Various studies have shown that AA and similar mutual aid groups benefit individuals by improving their mental well‐being and increasing their self‐esteem (Seebohm et al., 2013). These groups can benefit their members in terms of socializing, making new friends and receiving emotional support from friends (Yildiz & Duyan, 2022). AA members can also reduce feelings of isolation through the social relationships formed within the group and take positive steps towards recovery (Pettersen et al., 2019; Vigdal et al., 2022). AA provides a non‐clinical setting where individuals share their recovery stories and strive to rebuild their confidence, sense of belonging and social identity.

Furthermore, studies focusing on AA have revealed that positive behavioural changes have been observed in individuals who regularly participate in this group. Group members can cope with deprivation and increase their motivation to quit through mutual support and experience sharing (Kelly & Yeterian, 2011). In addition, researchers have shown that AA plays a role in reducing symptoms of depression (Kelly et al., 2010), establishing healthy social networks (Kelly et al., 2011) and developing self‐efficacy (Adelman‐Mullally et al., 2021). Nevertheless, several studies highlight certain structural and functional limitations of AA. In particular, the flexibility of AA's participation criteria and its organizational structure can result in shortcomings when accounting for individual differences (Witbrodt & Delucchi, 2011). Previous studies emphasize that the flexibility of AA participation criteria and AA group structure/functioning, and its weakness in considering individual differences, may be risky in some situations. Furthermore, the literature discusses concerns regarding its exclusionary nature towards professionals (Akın, 2017) and the potential for in‐group friendships to influence risky decision‐making, which may facilitate the transmission of alcohol use attitudes through ‘influence’ or ‘social contagion’ (Fujimoto & Valente, 2012; Kaner et al., 2022).

### Social Identity Theory (SIT) and recovery

In understanding alcohol misuse and recovery processes, SIT, which extends beyond individual biological and cognitive processes, has become an important framework in the addiction literature in recent years. Based on the pioneering work of Tajfel and Turner (1979), this theoretical framework proposes that individuals derive their sense of self from their membership in various social groups and obtain psychological meaningfulness, belonging and self‐esteem from these identities. Turner et al. (1987) further argued that a social identity is activated when individuals define themselves in terms of group categories (‘we’).

These social identities play a pivotal role in shaping individuals' psychological worlds and, beyond that, in determining their capacity to cope with stress, their access to social support networks and ultimately their overall health status (Cruwys et al., 2025; Haslam et al., 2009; Jetten et al., 2012). This multifaceted interaction offers a critical perspective, particularly in understanding the function of support groups within recovery processes. For instance, in groups such as AA, this dynamic enables individuals to define themselves as belonging to a recovery community, thereby facilitating a shift away from an entrenched ‘addict’ identity and towards the development of new, healthier social identities (Best et al., 2016).

The application of SIT to addiction recovery and recovery maintenance behaviours has gained central prominence over the last decade, driven by theoretical frameworks such as the social identity model of recovery (SIMOR) and the social identity model of cessation maintenance (SIMCM). This model conceptualizes recovery essentially as a process of social identity transition; in other words, as a transformation from an ‘addicted’ identity towards a ‘recovering’ identity. According to SIMOR, successful recovery entails three fundamental social identity processes: distancing from former identities and group memberships associated with substance use, acquiring new recovery‐oriented group memberships and internalizing and reinforcing these new identities within daily life (Best et al., 2016).

Conceptualized by Frings and Albery (2015), the SIMCM complements this framework by focusing on the cognitive mediators involved in sustaining the recovery process. The SIMCM emphasizes the central role of social identity transitions in cessation maintenance, suggesting that social identities operate at both explicit and implicit cognitive levels. According to this model, as long as individuals continue to identify with their former ‘user’ identity, the risk of relapse increases; conversely, the adoption of a new and positive social identity bolsters recovery. Research employing the SIMOR and SIMCM frameworks has demonstrated that social identity change among individuals in recovery is strongly associated with both reductions in substance use and improvements in quality of life (Best et al., 2016; Haslam et al., 2018; Lancaster et al., 2025). In particular, strong identification with a ‘recovering’ identity has been shown to be associated with aligning individuals' behaviours with the norms of the recovery group and enhancing self‐efficacy (Anderson et al., 2021; Best et al., 2016; Dingle et al., 2015).

Empirical findings from these theoretical models consistently demonstrate the decisive impact of social identity processes on recovery outcomes. Research reveals that individuals who attribute their recovery to social support and group memberships exhibit more sustained and enduring recovery compared with those who attribute it to individual achievement (Bathish et al., 2017). Similarly, active involvement in positive social identity groups (e.g., recovery communities and interest‐based groups) has been found to be associated with both a reduction in substance use and an increase in psychological well‐being (Buckingham et al., 2013; Haslam et al., 2019). These findings suggest that social identity processes play a critical role in addiction treatment, particularly within mutual aid groups.

### The present study

The role of social identity and group belongingness in the recovery process from AUD has been extensively documented in the existing literature. A robust body of qualitative and quantitative research has established that AA groups facilitate the transition from an ‘addicted’ identity to a ‘recovery’ identity (Best et al., 2016; Dingle et al., 2015; Frings & Albery, 2015). Empirical studies have demonstrated that identifying with a recovery group and internalizing its shared narratives support treatment retention and long‐term sobriety (Beckwith et al., 2015; Frings et al., 2019). While research conceptualizing these processes through the lenses of solidarity, fellowship and social support is prevalent (Bulumac, 2024; Frings et al., 2019; Glassman et al., 2020; Ratliff, 2003; Smith et al., 1993), the majority of existing work has primarily focused on the positive dimensions of identity transformation.

Despite this substantial foundation, empirical evidence remains limited regarding the negotiation processes involved in the construction of a recovery identity when confronted with in‐group threats such as boundary violations, vulnerabilities and relapse. Within the framework of SIT, this research aims to provide empirical evidence specifically within the Turkish context, moving beyond viewing AA merely as a supportive environment to examine how in‐group interactions actively produce a recovery identity and the threats to the maintenance of this identity. To this end, the study addresses the following research questions: (i) How does the AA group contribute to the members' sense of belonging and the construction of their recovery identity? (ii) Under what circumstances does the recovery identity become fragile, and how do members manage these threats?

## METHOD

A phenomenological approach was adopted in the study to gain an in‐depth understanding of individuals' experiences of the recovery process within the context of their social interactions. This approach enables participants to explore their personal experiences and worlds of meaning (Creswell & Poth, 2016), allowing individuals to focus on their experiences through their own narratives and understand social interaction patterns contextually.

### Participants

Participants were selected from among volunteers residing in Istanbul who regularly attended AA groups and maintained a relationship with this community for at least 6 months, using purposive sampling (Creswell & Plano Clark, 2011). In this study, ‘volunteer participants’ refers to active AA members who, after being informed about the aims of the research, confidentiality principles and ethical procedures, provided written and/or verbal consent to participate in face‐to‐face in‐depth interviews, attended AA meetings regularly and had maintained involvement with the AA community for at least 6 months.

AA meetings in Türkiye are held only in certain cities, and Istanbul is the city with the most meetings, with meetings taking place in eight districts. The city of Istanbul was selected to enable participation in multiple AA groups through maximum diversity sampling (Patton, 2015). Across multiple AA groups, 50 members were approached during open meetings. Of these, 25 agreed to receive detailed information regarding the study, and in‐depth interviews were subsequently conducted with 20 participants who met the inclusion criteria and volunteered for the study. Individuals who could not be interviewed were excluded for two primary reasons: (i) as the research design was based exclusively on face‐to‐face interactions, requests for online interviews could not be accommodated; and (ii) interviews could not be finalized due to mutual scheduling conflicts.

In order to reach participants, researchers attended various AA groups' open meetings as observers for specific periods of time, introduced themselves and established a relationship of trust. After the observer's participation, contact was established with the meeting participants, and those interested were informed about the research subject. The contact information of individuals who wished to participate in the study voluntarily was collected. Participants did not receive any financial or material compensation for their participation. The data collection process was terminated when no new information or themes emerged from the data obtained during the interviews, that is, when saturation was reached (Miles & Huberman, 1994). Saturation was reached when consecutive interviews yielded no new codes or insights (Guest et al., 2020). The sample size of 20 participants was sufficient to achieve analytical depth and thematic saturation within this relatively limited recovery community.

Interviews were conducted with 20 adults, including 10 women and 10 men, from three different AA groups. Gender balance was maintained within the participant group to capture both female and male experiences; however, gender was not defined as a predetermined analytical category in the research design. During the analysis process, as gender‐based dynamics became evident in the participant narratives, these themes were addressed through an inductive approach.

To protect participant anonymity within a small recovery community, specific identifiers such as occupation, relationship status and sobriety duration were aggregated when reporting demographic information (see Table 1). Looking at the demographic structure of the participant group, the ages of the participants ranged from 27 to 60. When examining educational levels, it is seen that the majority are bachelor's degrees (*n* = 12) and master's degrees (*n* = 6) graduates, two participants are high school graduates and one participant is a middle school graduate. The participants varied by occupation, including private sector employees, self‐employed individuals, public sector employees and students.

**TABLE 1:** Participants' demographic information.

	*n*	%
Gender		
Female	10	50
Male	10	50
Age group (years)		
25–34	3	15
35–44	7	35
45–54	7	35
55–64	3	15
Educational level		
Middle school	1	5
High school	1	5
Bachelor's degree	12	60
Master's degree	6	30
Marital status		
Married	5	25
Divorced	5	25
Single	10	50
Employment status		
Employed (full time)	19	95
Student	1	5
Frequency of meeting attendance (per week)		
1–2 days/week	6	30
3–4 days/week	7	35
5–7 days/week	7	35
Period of sobriety		
0.5–2 years	9	45
3–5 years	5	25
6+ years	6	30
AA sponsorship status		
Has a sponsor	19	95
Does not have a sponsor	1	5
Total	20	100

Abbreviation: AA, alcoholics anonymous.

The frequency of participants' attendance at AA meetings ranged from 1 to 7 days per week, and this regularity is important for understanding the dynamics of social support during the recovery process. The participants' periods of sobriety ranged from 6 months to 10 years.

### Data collection process

In this study, data were collected through semi‐structured, in‐depth interviews. The interview guide was developed by drawing on previous qualitative and theoretical studies regarding recovery, mutual aid groups and social identity processes while remaining sufficiently flexible to allow participants to raise issues they found personally meaningful. The interview questions were structured to encourage participants to express their experiences freely, rather than employing language that directly refers to theoretical concepts. These questions encompass thematic areas designed to elicit processes such as belonging, group membership, norms and identity transformation, drawing from participants' initial encounters with AA, their motivations for continued attendance, intra‐group relationships and recovery experiences. The complete set of interview questions and their analytical counterparts within the SIT framework are presented in Table 2.

**TABLE 2:** Alignment of semi‐structured interview questions with SIT.

No.	Interview question	Theoretical link to SIT
1	How did you start using alcohol?	Pre‐recovery context (not directly SIT related)
2	What happened in your life after you decided to quit alcohol?	Identity crisis/transition
3	How did you get involved with AA?	Entry into in‐group, social categorization
4	Can you talk about your experiences in the AA group?	In‐group norms, belonging, collective identity
5	What does AA mean to you?	Group identification, in‐group value
6	How did your social relationships change after joining AA?	Social identity continuity
7	What motivates you to continue attending AA?	Identity investment, in‐group commitment
8	Can you talk about your friendships in the AA group?	In‐group solidarity and mutual support
9	Is there anything else you would like to add about this process?	Open‐ended identity themes

Abbreviations: AA, alcoholics anonymous; SIT, social identity theory.

All interviews were conducted face‐to‐face in quiet and suitable locations in the neighbourhoods preferred by the participants, in accordance with the principles of confidentiality and voluntary participation. During and after each interview, the researchers took brief reflexive notes on contextual observations and emotional responses to maintain awareness of potential researcher bias throughout the data collection process.

Interviews were conducted between 9 September 2024 and 19 January 2025. The interviews lasted between 45 and 60 min. All interviews were conducted in Turkish, the participants' native language and the primary language used in AA meetings. This choice enabled participants to express their experiences comfortably and candidly. Transcripts were subsequently translated into English for publication, and the translations were collaboratively reviewed for accuracy and consistency of meaning. Interviews were transcribed verbatim in Turkish by the researchers before analysis.

### Data analysis

The audio recordings obtained from the interviews were transcribed into written text and analysed using MAXQDA (version 2024). The analysis followed Braun and Clarke's (2006) six‐phase framework, including familiarization with the data, generating initial codes, searching for themes, reviewing themes, defining and naming themes and producing the final report. As a result of the interviews, 263 pages of written text were reviewed multiple times to become familiar with the data. Meaningful statements were then identified, and initial codes were created. To enhance the reliability and credibility of the analysis, researcher triangulation was applied throughout the process (Patton, 2015). Four researchers independently coded the transcripts, subsequently holding consensus meetings to compare interpretations and reach an agreement on the themes. The themes were actively constructed through an iterative and reflexive analytical process.

The multi‐researcher triangulation process served as a form of peer review, enabling different perspectives to contribute to the validation of the themes. As a result of these discussions, the codebook was repeatedly reviewed and finalized. The coding process was conducted according to Saldaña's (2021) The Coding Manual for Qualitative Researchers, which provides a systematic framework for code development and refinement.

Given the interpretive nature of qualitative research, researchers kept reflexive journals to monitor their personal assumptions and emotional responses throughout the data collection and analysis process. None of the four researchers were AA members, which minimized internal bias, but all had prior academic or professional experience in the field of social work and addiction research. This background provided both familiarity with the subject matter and insight into participants' recovery experiences, contributing to a balanced interpretation of the data.

### Ethical issues

The ethical approval of this research was obtained from the Social and Human Sciences Research Ethics Committee of Istanbul University‐Cerrahpaşa (No. E‐74555795‐050.04‐1144057). Furthermore, the research process was conducted in accordance with the steps outlined in The Standards for Reporting Qualitative Research (SRQR) (O'Brien et al., 2014). All transcripts and audio files were stored on password‐protected devices accessible only to the research team. Participants were informed that the files would be destroyed within 3 years after the completion of the research, in accordance with the institutional data retention policy.

Participants' identity information was kept confidential during data transfer, and all data obtained were anonymized and used solely for scientific purposes. To ensure confidentiality, instead of participant names, female participants were assigned code names from ‘F1’ to ‘F10’ in order of appearance in the interviews, while male participants were assigned code names from ‘M1’ to ‘M10’.

## FINDINGS

### AA as an in‐group: The construction of a recovery identity

This theme demonstrates that participants' motivations for sustained attendance in AA are closely intertwined with the construction of a recovery‐oriented social identity. Participant narratives reveal that AA membership affords individuals the opportunity to join a group through a ‘recovering’ identity, providing a space where they experience a sense of belonging and can reconstruct their self‐esteem. For instance, F3 reported identifying herself through shared attributes with other AA members:

For me, AA is a social environment because, as I said, it is a community of people who are like me, who think like me, who are the same as me, who have the same illness. I feel incredibly good about that group. I feel like I am at home, like I am with my family. (F3, 52, 6 months sober)

AA membership also affords individuals the opportunity for future‐oriented identity construction. Members who have achieved long‐term sobriety serve as both a source of social comparison and a ‘possible self’ for participants, while shared narratives of failure reframe individual feelings of inadequacy as a collective experience:

In there [AA], I saw people who had succeeded. I mean, I saw people who had managed to stay sober for decades. I saw people who were able to quit, and I realized we had gone through similar processes. They, too, had tried to quit before AA and failed. So, I realized I wasn't alone. We had similar experiences of failure. My self‐confidence was at rock bottom. I arrived having completely lost my sense of self‐esteem. (M5, 52, 1 year 10 months sober)

Furthermore, participant accounts indicate that involvement in AA provides a departure from societal discourses that frame alcohol use as a moral failure. Redefining alcohol misuse as a shared illness rather than a ‘lack of willpower’ mitigates the internalized stigma of the participants and contributes to the construction of a recovery identity on a more acceptable and sustainable foundation:

They [those outside of AA] would say to me, ‘Look, I can have three drinks and stop, or four drinks and stop; why do you bring it home, why do you keep going, or why don't you just not drink at all?’ We weren't able to demonstrate that kind of willpower. But when I came here, what I was told was that this is a disease and that it had nothing to do with willpower. This provided such a sense of relief. That's when my self‐hatred began to fade. (M4, 38, 6 months sober)

Moreover, the sense of providing support to others within AA reinforces participants' legitimate standing within the group and can positively bolster the recovery identity. This dynamic can also be interpreted as the practical manifestation of the principle found in the 12 steps of AA, which emphasizes carrying the message to others as a result of a spiritual awakening:

‘Freely you received, freely give.’ When I first came here, people shared their experiences and insights with me without expecting anything in return, and that's what kept me sober. We must ensure this door remains open—even if it's just for one person—so that whoever comes in need will always find someone here. (M8, 36, 10 years sober)

### Reinforcement of the recovery identity through social interactions

This theme reveals that the sense of belonging is a social identity process actively enacted through recurring interactions and in‐group norms within AA. The fellowship in AA offers individuals an environment of unconditional acceptance, regardless of their past experiences, mistakes, social status or physical appearance. The narratives demonstrate that group norms, which subordinate differences in status and background, enable members to feel equal and accepted:

Everyone, once you get rid of your ego, once you realize that person doesn't have money, doesn't have a job, doesn't have a certain look, doesn't have whatever, whatever their past was, everyone accepts everyone else as they are. (M9, 39, 1 year 6 months sober)

Additionally, the friendship structure of the AA group offers an inclusive form of bonding that recreates a sense of belonging among its members. According to the participants' statements, even if they had been away for a long time or were joining a group for the first time, they were warmly welcomed and sincerely accepted. Recognition and a warm reception in new environments facilitate the participants' experience of AA identity as a portable and continuous social identity that transcends physical boundaries:

Brand‐new AA friends, valuable friends, have entered my life. These are extremely important. So when I go abroad [even], when I walk through the door of that AA meeting, and someone I don't know at all in that city asks my name and says, ‘Welcome [saying my name],’ and hugs me with a smile. (F5, 41, 2 years sober)

Friendships among AA members involve deep solidarity, nourished by a shared struggle. Most participants stated that sharing similar pain and recovery processes led them to describe their relationships as ‘fellow sufferers, shared pain’. The emphasis on shared pain and common experiences facilitates the recontextualization of individual life histories within a collective ‘we’ narrative:

So we socialize there, we build beautiful friendships. Everyone understands each other very well. We have been through similar experiences. We're soulmates. (F3, 52, 6 months sober)

Additionally, the friendships that develop among AA members are not limited to meeting times alone; they are strengthened by interactions before and after meetings, evolving into a social support network. Extra‐group interactions facilitate the translation of group norms into everyday life and bolster the continuity of the recovery identity:

I can have coffee with a female friend from AA. So, if there's something personal, I want to share, I can share it there too. Since we don't just share things at meetings, we're creating a support group for ourselves. (F4, 41, 6 years sober)

Furthermore, AA norms such as unconditional acceptance and the 24‐h rule provide a foundation for the continuous testing and validation of the individual's new identity. The unconditional acceptance highlighted by participants dissolves status differences, rendering the ‘recovering alcoholic’ identity as the superordinate identity and enhancing collective self‐esteem. For instance, the joy expressed by group members regarding sobriety creates positive group pressure, evolving into a motivational source that reinforces recovery behaviour:

There is such warmth, truly as if we are in the same struggle. I feel that they are happy. I mean, they are genuinely happy that I have not had a drink for twenty‐four hours. I feel the same way too. (M4, 38, 6 months sober)

M9's statement also underscores that AA discourses redefine individuals' relationships with alcohol through daily interactions. Consequently, as individuals identify more closely with a group, they appear to internalize that group's norms as a primary reference point for their own behaviour:

There is a saying that goes, ‘One drink is too many, and a thousand are not enough.’ I was very surprised when I heard this there because my own method did not include that, it was focused on moderation. It turned out that it was about quitting, quitting completely. After hearing those slogans, the impulse to drink was over for me. (M9, 39, 1 year 6 months sober)

These findings suggest that normative discourses in AA function as identity mechanisms that structure sobriety. Principles and slogans such as the 24‐h rule clarify boundaries and facilitate an abstinent self‐definition, serving as practical benchmarks for daily decisions. Consequently, sobriety becomes a shared object of collective commitment. Furthermore, extra‐group interactions ensure identity continuity by successfully translating these group norms into everyday life.

### Vulnerabilities and threats to the recovery identity

This theme reveals that despite AA membership providing a strong foundation for belonging, the recovery identity remains fragile and subject to constant negotiation. Participant accounts indicate that the recovery‐oriented social identity established through AA membership can be threatened by boundary violations and gender‐based interactions. Some participants expressed experiencing the healing and challenging aspects of close relationships within AA simultaneously, noting that intense emotional bonds with peers who relapse create contradictory experiences between a sense of belonging and personal vulnerabilities. From the perspective of SIT, an intense bond with a relapsing group member can threaten identity continuity by making it difficult for the individual to define themselves as a ‘recovering’ member. Consequently, the act of distancing reflects an identity management strategy developed in response to this threat:

I had a very close friend, and one day she went out drinking, and I was devastated. I struggled so much during that time while she was drinking. That's why there always must be a limit, because we're all alcoholics and we could all drink one day. (F2, 27, 1 year sober)

Certain discourses within AA, particularly the emphasis on self‐criticism and ‘character defects’, can manifest as emotionally taxing rather than supportive for some participants. According to the AA 12‐step program, ‘defects’ refer to personal flaws or negative traits (such as selfishness, dishonesty, resentment and pride) believed to contribute to addictive behaviours. Accordingly, these defects are expected to be identified, acknowledged and addressed as part of the recovery process (Steps 4–7 of the 12 steps). The implementation of this principle was observed to create a tension between AA group norms and the participants' need to maintain a positive self‐concept. While maintaining their group membership, participants managed this situation by selectively distancing themselves from specific normative components, such as the character defect discourse:

The constant labeling of oneself and the talk of character defects do not feel good to hear. Hearing those things is not helpful and that is why I am avoiding them to some extent. (M9, 39, 1 year 6 months sober)

Furthermore, it is observed that gender plays a decisive role in determining the quality and depth of social relationships. The narratives of female participants indicate that interactions established with same‐sex peers possess a more intimate, secure and supportive character:

I cannot establish the same level of intimacy with men. I feel much closer and warmer toward female alcoholics. We chat with them for 15 to 20 minutes every day and this feels as beneficial to me as attending a meeting. (F3, 52, 6 months sober)

These narratives suggest that same‐sex relationships provide a form of identity security for female participants. Women reported being able to share experiences that are difficult to articulate in mixed‐gender groups, such as sexual harassment and domestic violence, more comfortably in women‐only AA meetings:

Women‐only meetings are much more private. They offer an environment where we share our intimate experiences more deeply. A woman understands another woman better. (F6, 37, 2 year 5 months sober)

Interactions with the opposite sex in AA reveal potential risks that could disrupt the recovery process due to the possibility of emotional involvement and the blurring of interpersonal boundaries. In this context, the frequently used ‘hedgehog metaphor’ stands out as a meaningful expression that draws attention to the balance between distance and trust in relationships:

We are like hedgehogs. If we don't snuggle up to each other at night, we get cold, but if we snuggle too close, our quills prick each other. (F9, 29, 1 year 6 months sober)

This metaphor illustrates how participants maintain both group identity and personal identity continuity by establishing strategic closeness instead of opting for total isolation or complete fusion. Furthermore, it is observed that a significant dimension of women's experiences within AA is shaped by gender‐based judgements. Particularly, power asymmetries and moral judgements arising in male–female relationships can lead female participants to limit their in‐group behaviours. Being a woman with alcohol addiction, when combined with sexist stereotypes, often results in stigmatizing perceptions such as immorality or unreliability:

Nowadays, being a woman who drinks heavily also means being promiscuous. It also means having a criminal record and being documented. Some people in the AA community have this bias. Having a decent marriage does not prevent people from looking at you askance. Because you are an alcoholic, you could cheat on your husband, you could sleep with whoever they want or whoever you want. (F1, 50, 6 years sober)

## DISCUSSION

The findings of this study reveal that AA provides an interactional group context where a recovery‐oriented social identity is constructed, negotiated and maintained for its members. Furthermore, the results demonstrate that AA membership facilitates a departure from stigmatized identities associated with AUD, creating a new space of belonging centred on the identity of a ‘recovering person’. This identity transition is addressed in the literature regarding its capacity to facilitate the recontextualization of the past within a new recovery‐oriented belonging and the construction of a more coherent future‐oriented self‐narrative (Best et al., 2016; Dunlop & Tracy, 2013). Indeed, our findings are consistent with the literature in supporting that belonging to a recovery group yields positive outcomes (Cruwys et al., 2020; Frings & Albery, 2015) and serves a relapse prevention function throughout the transition from an addicted identity to a recovery identity (Buckingham et al., 2013; Dingle et al., 2015; McIntosh & McKeganey, 2000).

Another fundamental feature of AA groups that facilitates recovery is that members listen to and support one another without judgement. This stems from the fact that personal stories shared during AA group meetings foster empathy and mutual understanding among members (Abu Hassan Shaari & Waller, 2023; Humphreys, 2000). This environment provides members with the opportunity to express themselves within a secure setting devoid of the fear of exclusion or criticism stemming from past mistakes or AUDs. Viewed through the lens of SIT, this non‐judgemental context establishes an identity security space that allows individuals to exist within the group without being subjected to identity threats. Haslam et al. (2018) emphasize that social identities can function as protective factors for health and well‐being, though this is only achievable when the group context provides psychological safety. The structured meeting format and the principle of anonymity in AA stand out as key structural features that facilitate this secure environment.

Furthermore, the presence of senior members who have achieved long‐term sobriety in AA strengthens the sense of collective efficacy by functioning as prototypical figures and possible selves for newcomers. Within the context of AA, the presence of members with long‐term sobriety provides concrete evidence that recovery is possible, thereby bolstering the self‐efficacy beliefs of new members (Bulumac, 2024; Islam et al., 2023). This process enables the individual to transition from a socially marginalized position into a psychologically safe in‐group space defined by a shared fate. Consistent with findings in the broader literature, this process of self‐categorization and group identification strengthens collective efficacy and enhances motivation for recovery (Best et al., 2016; Buckingham et al., 2013). Frings and Albery (2015) interpret the impact of possessing a positive collective self‐esteem as the indirect reflection of group success onto each individual and the ability of group members to benefit from the achievements or status of their peers, and the findings of this study point to a similar process of social identity reflection.

The literature indicates that helping behaviour in AA is an important factor supporting the individual recovery process (Acar et al., 2022; Groh et al., 2008; Zemore et al., 2004), and the findings of this study also show that the desire to help others is a fundamental motivational source for individuals to maintain their commitment to AA. Previous research in the literature also emphasizes that group members are more likely to help others with whom they share a common identity and that this behaviour reinforces group belonging (Gray & Stevenson, 2020; Hughes et al., 2024). From the perspective of SIT, helping others within AA reflects the internalization of the collective identity of recovery; as members identify with the group, supportive behaviours towards other members become an expression of a shared self‐definition, solidarity and a sense of responsibility towards the group (Abu Hassan Shaari & Waller, 2023; Buckingham et al., 2013; Leach et al., 2008). This process can also be conceptualized as an interactional one whereby shared expectations and norms regarding the behaviours of those in recovery are constructed and reinforced through communication within small group interactions (Postmes et al., 2005).

Normative discourses in AA, such as ‘staying sober for twenty‐four hours’ and ‘one drink is too many’, delineate the prototypical boundaries of the recovery group. This process facilitates participants' self‐stereotyping in accordance with the definition of a ‘typical recovering person’, thereby enabling the construction of the group as a more homogeneous ‘us’. At the same time, these norms transform sobriety into a shared value and an object of commitment, thereby strengthening the sense of solidarity, satisfaction and centrality towards group membership. This bidirectional process is consistent with the framework of Leach et al. (2008), which conceptualizes in‐group identification through the dual dimensions of self‐definition and self‐investment. Best et al. (2016) argue that in the construction of a recovery‐oriented social identity, group memberships exert influence on individuals through the transmission of social norms that are internalized and shape attitudes and behaviours, thereby increasing the likelihood that group norms will be integrated into the individual's own sense of self.

In this context, AA membership is redefined as a form of social identity that extends beyond the group environment into daily life practices, as demonstrated by our findings. Participants establish a new social network with peers in recovery. Previous research has highlighted that the friendships formed among AA members have positive effects on the likelihood of maintaining abstinence (Longabaugh et al., 2010; Wnuk, 2022). The results of our study align with the literature, suggesting that these newly formed social resources contribute to the recovery journey and the internalization of a sense of belonging within everyday life (Blondé et al., 2024; Cruwys et al., 2020; Taylor et al., 2020). These findings indicate that social identity becomes embodied through concrete social relationships and networks.

One of the most striking results of the study is the fragile nature of the recovery identity and its negotiation with threats. Specifically, the relapse of a member with whom a close bond has been established is experienced as an ‘in‐group identity threat’ that jeopardizes the collective success of the group and the individuals' perceived identity continuity. This finding can be explained through the mechanisms of in‐group differentiation and subtyping in the social identity literature (Marques et al., 1988). Marques et al. (1988) demonstrated that group members react more negatively to in‐group members who violate normative expectations compared with out‐group members, a phenomenon known as the ‘black sheep effect’. Within the context of AA, the distant stance towards members who experience relapse can be interpreted as an attempt to reaffirm the boundaries of the recovery identity.

This situation can be interpreted as some AA members framing recovery as a gradual transition from ‘user groups’ to ‘recovery‐supporting groups’ and redefining the distance maintained with individuals who continue alcohol use (De Meyer et al., 2025). The ‘hedgehog metaphor’ employed by participants reflects the dilemma where social bonds represent both a source of recovery and a risk of injury. This dynamic necessitates a strategic relational distance that individuals establish with group members to safeguard their recovery identity. From the perspective of the social identity approach, these findings reveal the complexity and contextual variability of identity management strategies (Tajfel & Turner, 1979). Furthermore, Glassman et al. (2022) highlighted the flexible structure of AA and demonstrated that various facets of the program can be negotiated and adapted at an individual level.

In the Turkish context, the moral scrutiny and stigma that women encounter due to alcohol misuse (Atlam et al., 2024; Gürel et al., 2025) emerge as one of the primary cultural barriers to the development of a recovery identity. Consistent with previous research in different national contexts such as Brazil (Antunes De Campos et al., 2023) and other existing studies (Kelly & Hoeppner, 2013; Kornfield, 2014; Sanders, 2019), this investigation reveals that women are subjected to gender bias and social stigmatization in mixed‐gender AA meetings. A prominent finding of this study is that identity threats associated with stigma and shame can be mitigated through women‐only AA meetings, which provide essential support for maintaining a collective recovery identity.

In conclusion, this study demonstrates that within the Turkish context, AA membership reveals recovery to be a collective experience structured through social identities rather than a process of individual willpower. The findings illustrate that social identity is constructed, maintained and at times rendered fragile within small group interactions, thereby providing a contextual and qualitative contribution to SIT and related perspectives.

### Limitations and recommendations for future research

This study is subject to several limitations. The participants are restricted to AA groups located in Istanbul, and the findings are not intended for direct generalization to the diverse urban settings and group dynamics across Türkiye. Moreover, since social and cultural variables such as ethnicity and religious practices, alongside recovery‐related factors like the severity of alcohol use and duration of membership, were not systematically collected, the correlation between these dimensions and identity processes within AA could not be directly examined. Lastly, because participation was voluntary, there is a possibility that individuals with stronger attachments to AA are overrepresented in the sample. Future studies could explore the heterogeneity of identity processes more thoroughly by specifically focusing on the experiences of members who exhibit weaker identification or irregular attendance.

## CONCLUSION

This study provides qualitative evidence that recovery identities in AA are continuously negotiated processes shaped by daily interactions, group norms and structural vulnerabilities. In our research, AA functions as an in‐group where members' narrative practices construct a shared recovery identity through mutual support. Simultaneously, this recovery identity is extended beyond the group as social networks composed of group members are transposed into daily life practices. Furthermore, the findings expand social identity research in addiction recovery by demonstrating the existence of structural constraints on identity negotiation within mutual aid groups.

## AUTHOR CONTRIBUTIONS

**Fatih Kucur:** Conceptualization; methodology; data curation; supervision; formal analysis; writing – review and editing; writing – original draft; investigation; validation. **Özge Kelebek:** Conceptualization; methodology; investigation; writing – original draft; writing – review and editing; formal analysis; validation. **Melek Rabia Kapıcı:** Methodology; data curation; writing – original draft; validation. **Esme Temiz:** Methodology; data curation; writing – original draft; validation.

## FUNDING INFORMATION

This research did not receive any specific grant from funding agencies in the public, commercial, or not‐for‐profit sectors.

## CONFLICT OF INTEREST STATEMENT

No potential conflicts of interest were reported by the authors.

## ETHICS STATEMENT

The ethics approval of this research was obtained by the Istanbul University‐Cerrahpaşa Social and Human Sciences Research Ethics Committee (Number: E‐74555795‐050.04‐1144057).

## INFORMED CONSENT

Informed consent was obtained from all individual participants included in the study.

## Data Availability

Data associated with this paper are potentially identifiable. The data are only accessible by the research team, as a requirement of the ethics approval for the study, to protect the confidentiality of the participants.
